# Effect of melatonin on insomnia and daytime sleepiness, in patients with obstructive sleep apnea and insomnia (COMISA): A randomized double-blinded placebo-controlled trial

**DOI:** 10.1186/s40780-024-00347-9

**Published:** 2024-05-30

**Authors:** Tahereh Madani Motlaq, Besharat Rahimi, Shahideh Amini

**Affiliations:** 1 Department of Clinical Pharmacy, Faculty of Pharmacy, Tehran University of Medical Sciences, Tehran, 1417614411 Iran; 2 Research Center for Rational Use of Drugs, Tehran University of Medical Sciences, Tehran, 1417614411 Iran; 3 Advanced Thoracic Research Center, Tehran University of Medical Sciences, Tehran, Iran; 4 Rajaie Cardiovascular Medical and Research Institute, Tehran, 1995614331 Iran

**Keywords:** Melatonin, Obstructive sleep apnea, Insomnia, Comorbid insomnia, And obstructive sleep apnea (COMISA)

## Abstract

**Background:**

COMISA is a common disorder that results in nighttime awakenings ,daytime sleepiness and PAP intolerance. Cognitive behavioral therapy for insomnia is used to improve PAP adherence and no medication has been evaluated in such population yet. Melatonin with its chronobiotic and antioxidant effects may have potential benefits on COMISA consequences at the appropriate dose and time. This study aimed to evaluate the effect of melatonin on sleep quality, daytime sleepiness and PAP Compliance in patients with COMISA.

**Methods:**

This double-blind placebo trial randomly assigned eligible OSA patients who suffered from insomnia despite using PAP for over a month to receive either melatonin 10 mg or placebo. The primary outcomes were measured by changes in the Pittsburgh Sleep Quality Index (PSQI), Insomnia Severity Index (ISI), Epworth Sleepiness Scale (ESS), and Functional Outcomes of Sleep Questionnaire (FOSQ-10) over one month. Adherence to PAP was measured by the results of the PAP device reports on the average length of time and number of nights that the device was used.

**Results:**

Thirty patients were enrolled in the study after randomization. The melatonin arm showed significant improvement in all four primary outcomes compared to the placebo arm. The PSQI score was 3.836±1.839 in the melatonin arm versus 10.522±3.626 in the placebo arm (*P*value<0.001). The ISI score was 8.476±3.568 in the melatonin arm versus 14.47±4.50 in the placebo arm (*P*value<0.001). The ESS score was 6.854±4.334 in the melatonin arm versus 13.298±5.119 in the placebo arm (*P*value<0.001). The FOSQ-10 score was 24.93±5.02 in the melatonin arm versus 19.87±4.24 in the placebo arm (*P*value= 0.006). Additionally, nighttime consequences such as sleep latency and awakenings showed significant improvement in the melatonin arm. PAP devices results revealed improvement in duration of PAP use overnight.

**Conclusions:**

Administering melatonin has been shown to improve self-reported sleep quality and PAP adherence in patients with COMISA.

**Trial registration:**

Registration number IRCT20220105053635N1 was issued by the Iranian Registry of Clinical Trials (IRCT).

## Background

Obstructive Sleep Apnea (OSA) and insomnia are highly prevalent sleep disorders, commonly present simultaneously. The prevalence of comorbid insomnia with obstructive sleep apnea (COMISA), has been estimated 10.7 to 88 percent due to variations in the population and diagnostic methods [1]. There is a bi-directional relationship between OSA and insomnia, which can describe this high prevalence [2]. Coexistence of two diseases, intensifies the burden of illness severity [3, 4]. Furthermore, insomnia and OSA share similar symptoms and risk factors. Patients experience decreased quality of life, mood disturbance, memory impairment, and daytime sleepiness, due to unrefreshing sleep and frequent awakening [5, 6]. Studies show that COMISA patients represent more sleep disturbance, cardiovascular diseases such as hypertension, cerebrovascular diseases, and diabetes [5, 7].

Since OSA contributes to insomnia, treating it with Continuous Positive Airway Pressure (CPAP) devices, could improve persistent insomnia. However the pathophysiological mechanisms of the two conditions are different, and a distinct approach is needed to treat COMISA. It showed that people may still experience daytime sleepiness and low subjective sleep quality, overnight. Moreover, Patients have poor adherence to PAP devices as an effective treatment of moderate to severe OSA compared to the OSA alone, in the first six months of usage [8]. Cognitive behavioral therapy for insomnia (CBTi), as a non-pharmacological treatment, was effective in patients with COMISA. Yet, improving sleep quality and increasing adherence to CPAP was not found in all of the patients and it was similar to the patients with insomnia alone [9–11].

Although nonpharmacological therapies are preferred and more effective, in patients who have not been treated with CBTi or were reluctant to receive it, short-course of doxepin, Z-drugs, and benzodiazepines, are recommended. However, they were not effective enough to increase sleep efficiency [12, 13]. Therefore, alternative options can be considered.

The Pineal gland-secreted hormone, Melatonin, shows a soporific and chronobiotic effect and has sleep-promoting properties. It can probably be effective in primary and secondary sleep disorders. Reviews revealed that melatonin may increase sleep quality and decrease sleep latency, in primary insomnia [14, 15]. Although secondary insomnia seems to be more difficult to treat, it is found that melatonin usage improves total sleep time, in these patients [15].

Melatonin has been used in comorbid insomnia, with various medical conditions [16]. Studies support this notion that either immediate or prolonged release of melatonin at the dose f 1-10 mg, not only can be prescribed safely, but also can improve sleep quality, sleep latency, and day- time sleepiness in patients with neuropsychiatric disorders like Parkinson's disease, respiratory disorders such as Chronic Obstructive Pulmonary Diseas (COPD), and sulfur mustard lung injuries [17–25].

According to studies, OSA and insomnia co-occurrence leads to more sleep complaints, than each of them alone. Besides, insomnia can exacerbate OSA and decrease PAP tolerance, and there is a lack of evidence in the selection of appropriate medication to improve sleep quality. Moreover, melatonin secretion alters in 25% of patients suffering from obstructive sleep apnea in a way that the peak level of melatonin at night decreases significantly, and the phase- shifting occurs. Besides, melatonin is effective in primary and secondary insomnia, due to its chronobiotic effect. Based on clinical trials and reviews, melatonin can have benefits in sleep quality, sleep efficacy, and sleep latency, in chronic and comorbid insomnia without any negative effects on respiration [26]. we hypothesize that melatonin could be an appropriate alternative to improve the sleep quality and daytime sleepiness, in patients with COMISA. Therefore, in this study, the efficacy of immediate release of melatonin 10 mg on subjective sleep quality and PAP adherence in the COMISA population, was investigated.

The current study contributes to the state of knowledge both in terms of theory and practice, (a) The effect of melatonin in COMISA was an extrapolation of studies on chronic insomnia. There was a lack of evidence of pharmacotherapy in such population. Moreover, the dose of melatonin was much more than the usual doses used in chronic insomnia, since melatonin at a dose of more than 10 mg has a longer half-life in the blood. So, mid-night waking would be covered. (b) Instruments and questionnaires used in this study were clinically useful so that they can easily apply in sleep clinics to diagnose COMISA. (c) Participants were monitored for adverse effects during and after the intervention and the causality was evaluated by the Naranjo score.

## Methods

### Participants

Patients with baseline moderate-severe OSA (Apnea-hypopnea Index (AHI) ≥15), aged 18-75 years old, who were treated for OSA using a CPAP device for at least one month, and had insomnia complaints, were considered eligible. Insomnia was diagnosed by The International Classification of Sleep Disorders – Third Edition (ICSD-3) diagnostic criteria and ISI (Insomnia Severity Index) scale. Patients with ISI scores more than 15 or more than 6 in the first three questions, were also eligible.

PSQI (Pittsburgh Sleep Quality Index) ≥ 5 showed poor sleep quality and was considered as an inclusion criterion.

After baseline assessment, the exclusion criteria were considered as following: severe pulmonary diseases (COPD GOLD IV, severe uncontrolled bronchiectasis, ILD, and asthma), severe heart diseases (NYHA class IV and uncontrolled hypertension), neurologic disorders (Parkinson disease and Alzheimer's disease beyond stage III based on Hoehn & Yahr stage scale and Global Deterioration Scale respectively), psychiatric disorders (using drugs, psychiatric disorders at least for 3 months, and bipolar disorder), using a medication affecting sleep and respiration (benzodiazepins, Z-drugs, neuroleptics), being allergic to melatonin, pregnancy and lactation, hypothyroidism, narcolepsy, restless leg syndrome, and being a shift worker. Besides, if the tablets were not taken for 4 days, the patient was excluded from the study.

### Study design

This study was a randomized, double-blind clinical trial, conducted in the sleep clinic of Emam Khomeini Hospital, Tehran, Iran. The forthcoming clinical trial was carried out adhering to the standards of the International Conference on Harmonization of Good Clinical Practice (ICH-GCP), the ethical principles of the Helsinki Declaration 2013, the general guidelines for ethics in human subject research in the country, the ethical guidelines for clinical trials in the country, and other national and university laws and regulations. Informed consent form and all questionnaires provided to all participants has been accepted by Tehran University of medical sciences ethics committee and this committee authorized this study (IR.TUMS.TIPS.REC.1400.177). Registration number IRCT20220105053635N1 was issued by the Iranian Registry of Clinical Trials (IRCT) on 19/04/2022.

### Randomization and blinding

After the eligible participants enrolled in the study, they were randomly assigned to the melatonin or placebo group. Random numbers of envelopes were prepared by a separate staff, not involved in assigning and assessment of patients, using an online randomization generator (www.sealedenvelopes.com). Random four blocks were used. Opaque-sealed sequentially numbered envelopes were used for allocation concealment. The envelopes and the tablets were similar in terms of color, shape and size, so all the people including researchers and patients, were blinded through the study.

### Study instruments

A questionnaire was filled out at the time of enrollment, providing demographic information as follows: age, weight, height, BMI (Body Mass Index), marital status, education, job, PMH (Past Medical History which includes cardiovascular disorders, kidney diseases, pulmonary disorders, psychiatric disorders, and rheumatologic disorders), using medications associated with sleep disorders, baseline AHI, and duration of PAP therapy.

Insomnia severity was assessed by the ISI scale. ISI is a seven-item questionnaire that assesses nighttime and daytime consequences due to insomnia, during the last two weeks. The scores for each question were added- up to get the final score. The categorization of patients for insomnia was based on the total score. A score below 7 showed no clinically significant insomnia, scores ranged from 8-14, represented subthreshold insomnia, and scores more than 15, were considered as moderate to severe insomnia. The first three questions assess difficulty in falling asleep, maintaining sleep, and problems waking up too early. An ISI score of more than 6 for these first three questions, can probably help diagnose co-morbid insomnia [1].

Sleep quality was assessed by PSQI (Pittsburgh Sleep Quality Index) questionnaire. It is a self-reported instrument for the assessment of sleep quality and sleep disturbance in patients, over a one-month interval. This scale assesses 7 components: a)Total sleep quality, b) Sleep latency, c) sleep duration, d) sleep efficiency, e) sleep disturbance, f) use of sleep medications, and g) next morning dysfunction. The score of each component is ranged from 0 to 3. The total score resulted from adding the score of each component. A total PSQI score of more than 5, was eligible to enroll in the study.

ESS (Epworth Sleepiness Scale) is a sleep test questionnaire that assesses daytime sleepiness, in eight daily situations. The ESS score of less than 7, shows no sleepiness. A score between 8-9 represents the average amount of daytime sleepiness. A score of more than 10, shows excessive daytime sleepiness.

FOSQ-10 (Functional Outcomes of Sleep Questionnaire) includes 10 questions presenting day -time dysfunction, resulting from insomnia in 10 different situations. It shows the effect of poor sleep quality on daily activities, memory, and concentration. The total score is ranged from 10 to 40. The FOSQ-10 score below 15, shows daily activity disturbance in patients. Besides, Higher scores represent better daily functions.

All Persian versions of the questionnaires were checked for validity and reliability [27–30].

For the assessment of adverse effects, a questionnaire was filled out, which was about the type of adverse effects (Headache, Confusion, Dizziness, Somnolence, Nausea, Fatigue), frequency, date of adverse effects happening, and time of recovery. The causality was assessed by the Naranjo score.

### Trial treatments

Prior to this, participants were taught the principles of sleep hygiene. Following an initial assessment, eligible patients were asked to complete an informed consent form before being randomly assigned to either the placebo or melatonin group. Patients in the melatonin group received 10 mg melatonin tablets (produced by Jalinous company), 30- 60 minutes before sleep, for one month. Placebo tablets which were quite similar to the melatonin tablets in terms of color, shape, and taste, were given to the patients in the placebo group in the same order.

If a participant experienced poor sleep quality during the study despite receiving treatment appropriately, trazodone tablets were administered at a dose of 25-50mg. This was done to ensure ethical treatment of patients suffering from insomnia. The date, number of pills, and dose of trazodone usage were recorded. Although the data on Trazodone usage in obstructive sleep apnea is limited, studies and reviws showed that trazodone had no significant effect on Sleep Apnea Severity and Architecture include nadir oxygen saturation and duration of respiratory events. Moreover, trazodone at the dose of 100 mg could increase arousal thershold without impairing pharyngeal dilator muscle function and reduces the AHI. That is the resone of choosing trazodone in such patients.

The study's primary outcomes were improvements in sleep quality and daytime sleepiness, as assessed by four questionnaires: the ISI, PSQI, ESS, and FOSQ-10. Participants completed these questionnaires at both the baseline and the end of treatment [31, 32].

The study assessed adherence to PAP devices as a secondary outcome by measuring the number of days and hours patients used the device appropriately. This was defined as using the device for more than 4 hours and on at least 70% of days in the month prior to study enrollment. The data was extracted from reports generated by the PAP devices both at the baseline and at the end of the treatment.

The patients' medication and PAP device adherence, as well as their adherence to sleep hygiene principles, were re-evaluated after 15 days and at the end of the study. The same questionnaires were used to assess sleep quality, insomnia severity, daytime sleepiness, and impaired daily function.

The safety analyses included all patients who received therapy. Common adverse effects such as hypersensitivity reactions, headache, confusion, dizziness, somnolence, nausea and fatigue were evaluated during the study almost every 7 days and one month after the treatment was finished. The Naranjo score was used to evaluate causality for any adverse effects and the form was filled at the end of the treatment period. Patients with severe adverse effects were excluded. Unblinding would be performed for all patients if adverse effects occurred in more than 12.5% of participants (at least 7) or if they were caused by melatonin use. Serious adverse effects resulted in immediate unblinding for both the participants and the researchers.

### Statistical analysis

Since there was lack of previous studies based on Iran in this population, the sample size was calculated according to other studies on comorbid insomnia and respiratory disorders [33], considering the error rate of 5%, power of 80%, α= 0.05 and β= 0.9 and using the following formula, a 50-subject sample size (25 in each group) was consider for this study:

$$n\frac{2\sigma^2\left(Z\alpha/2^{Z\beta}\right)^2}{\left(\mu_t-\mu_c\right)^2}$$

IBM SPSS version 27 was applied for statistical analysis. Kolmogorov-Smirnov and Shapiro–Wilk tests were performed to examine the normality of distribution. Chi-square is performed to find out independency. Mean and standard deviations expressed continuous data and categorical data were represented by numbers and percentages. Independent sample t-test and Mann–Whitney was used to find out the differences between two arms, for normal and non-normal distribution data respectively and P-value less than 0.05 was considered as significant. The effect size was examined by Cohen's. One and two-way ANCOVA tests were performed to eliminate the covariates' effect.

## Results

**Fig. 1:**
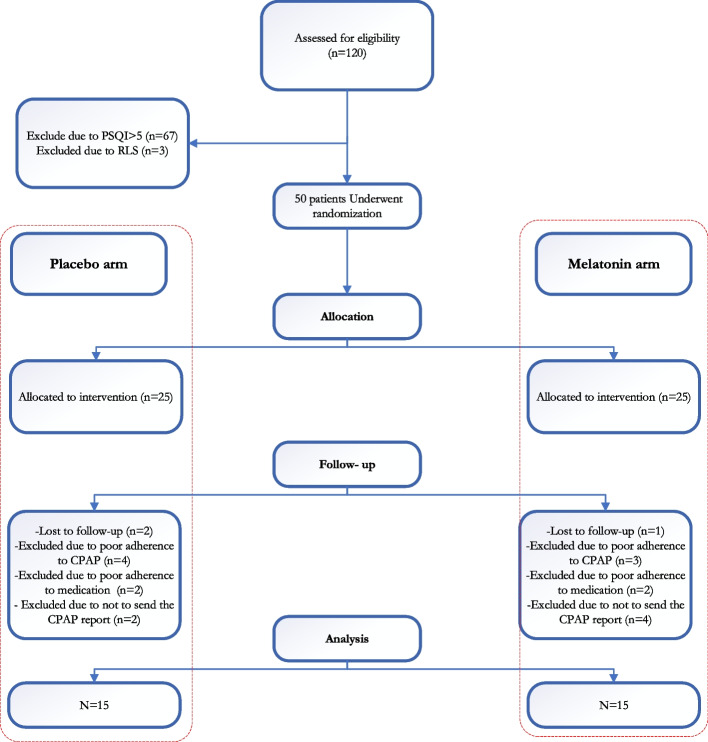
Flow chart of Randomization and Follow-up of the Patients

Among one hundred and twenty patients who underwent initial eligibility assessment, seventy patients were excluded due to not meeting the inclusion criteria. Thirty randomized participants completed the study. Figure 1 shows a flowchart of randomization and follow-up based on CONSORT 2010 guideline. Individuals were predominantly middle-aged (mean [SD] age, 58.53 [12.58], years) and female (70%). There was no significant difference in demographic features between two groups. The demographic characteristics of participants are presented in Table 1.

**Table 1:** Characteristics of the patients at baseline

**Main feature**	**Sub-feature**	**Number of patients**			
		**Total** **(*n*=30)**	**Melatonin (*n*=15)**	**Placebo (*n*=15)**	***P* value**
**Gender**	Male	9 (30)	6 (40)	3 (20)	0.232
	Female	21(70)	9 (60)	12 (80)	
**Age - no (%)** **(Mean± SD)**	<50 y.o	3 (10)	2 (13.3)	1 (6.6)	0.383
	≥50 y.o	27 (90)	13 (86.6)	14 (93.3)	
**BMI- no (%)**	<30	9 (30)	6 (40)	3 (20)	0.519
	30-40	15 (50)	7 (46.6)	8 (53.3)	
	≥40	6 (20)	2 (13.3)	4 (26.6)	
**Education- no (%)**	Illiterate	1 (3.3)	0	1 (6.6)	0.558
	<Diploma	14 (46.6)	7 (46.6)	7 (46.6)	
	Diploma	10 (33.3)	5 (33.3)	5 (33.3)	
	Bachelor	4 (13.3)	3 (20)	1 (6.6)	
	Dr	1 (3.3)	0	1 (6.6)	
**Marital status- no (%)**	Single	2 (6.6)	1(20)	1(20)	1.000
	Married	28 (93.3)	14 (80)	14 (80)	
**Past medical history- no (%)**	Non	7 (23.3)	4 (26.6)	3 (20)	0.545
	1-2 diseases	19 (63.3)	10 (66.6)	9 (60)	
	≥ 3 diseases	4 (13.3)	1 (6.66)	3 (20)	
**PAP usage duration- no (%)**	1-6 months	13 (43.3)	3 (20)	10 (66.6)	0.068
	7-12 months	2 (6.6)	2 (13.3)	0	
	13-24 months	4 (13.3)	2 (13.3)	2 (13.3)	
	>24 months	11 (36.6)	8 (53.3)	3 (20)	
**Hypnotic usage history- no (%)**	Non	26 (86.6)	13 (86.6)	13 (86.6)	
	BDZ	3 (10)	1 (6.6)	2 (13.3)	0.513
	Trazodone	1 (3.3)	1 (6.6)	0	
**Device type- no (%)**	CPAP	20 (66.6)	10 (66.6)	10 (66.6)	0.721
	Auto-PAP	4 (13.3)	2 (13.3)	2 (13.3)	
	BiPAP	6 ( 3.3)	3 (20)	3 (20)	
**Primary AHI- no. (%)**	15-30 events/hr.	8 (26.6)	5 (33.3)	3 (20)	0.463
	>30 events/hr.	22 (73.3)	10 (66.6)	12 (80)	

### Primary outcomes

Response in the placebo vs. treatment group was evaluated by four questionnaire scores (PSQI, ESS, ISI, FOSQ-10). Meaningful improvements in primary outcomes were found in the melatonin arm, in comparison to the placebo arm (Table 2, *P*value<0.001). Sleep quality measured by PSQI, demonstrates a significant difference pre- and post-intervention in the melatonin arm, in comparison to the placebo arm (2.481, [95% CI, 1.430 to 3.511] vs. 0.274, [95% CI, -.246 to 0.786], *P*value<0.001). Moreover, the severity and impact of insomnia assessed by ISI, improved significantly in the melatonin group versus placebo (1.679,[95% CI, 0.870 to 2.464] vs. 0.550,[ 95% CI, -0.003 to 1.086], *P*value<0.001). According to ISI scaling, patients in the melatonin arm were categorized as moderate to severe insomnia (mean[SD], total score, 15.93±5.788), which was changed to subthreshold insomnia (mean[SD], total score, 8.476±3.568), post-intervention. Whereas, in the placebo arm, patients suffered from severe insomnia (mean[SD], total score, 17.27±5.77), which improved to moderate insomnia (mean[SD], total score, 14.47±4.50).

**Table2:** Primary outcomes results

	**Arms**						**Mean Difference (SD) (CI 95%)**	***P*value**^**1**^	**Sample power**	**Effect size (95% CI)**^**2**^
**Primary outcomes**	**Melatonin (Mean±SD)**			**Placebo (Mean±SD)**						
	**Before**	**After**	***P* value**^**3**^	**Before**	**After**	***P* value**^**3**^				
**ISI- total score**	15.93±5.788	8.476±3.568	<0.001	17.27±5.77	14.47±4.50	0.051	-6.53 (1.48, -9.57 to - 3.49)	<0.001	0.998	-1.643 (-2.466, -.798)
**PSQI- total score**	8.764±3.019	3.836±1.839	<0.001	11.14±3.582	10.52±3.62	0.196	-6.53 (1.101, -8.78 to -4.27)	<0.001	1.000	-2.325 (-3.251, -1.375)
**ESS- total score**	11.93±4.935	6.854±4.334	<0.001	13.87±4.83	13.29±5.11	0.05	-6.467 (1.705, -9.960 to-2.974)	<0.001	0.95	-1.359 (-2.147, -.550)
**FOSQ-10- total score**	18.33±4.254	24.93±5.02	<0.001	18.13±4.22	19.87±4.24	0.009	5.06 (1.69, 1.59 to 8.54)	0.006	0.9	1.090 (0.312, 1.852)

ISI score represents the severity of insomnia. Scores more than 15 or more than 6 for the first three questions show insomnia. The sleep quality was measured by PSQI. The higher the PSQI score, the worse the sleep quality. ESS scoring indicates day-time sleepiness the scores more than 6 show day-time sleepiness. The daytime function is measured by FOSQ-10. Scores less than 15 indicate day-time function disturbance. The effect size based on Cohen's d shows a strong difference between the two means and improvement in the melatonin arm

^1^*P*value between groups, ESS and PSQI *P*value were assessed by Mann–Whitney, ISI and FOSQ-10 changes were assessed by independent sample T-test

^2^The effect size is based on Cohen's d

^3^*P*value within the group, ESS and PSQI Pvalue were assessed by Wilcoxon test, ISI and FOSQ-10 changes were assessed by Paired T-test

Daytime sleepiness score also decreased in the melatonin group in comparison to placebo, due to ESS scale (1.743,[95% CI, 0.916 to 2.546] vs. 0.232,[95% CI, -0.285 to 0.741], *P*value<0.001). Patients in both arms suffered from excessive daytime sleepiness (mean[SD], total score, melatonin arm, 11.93±4.935 vs. mean[SD], total score, placebo arm, 13.87±4.83). ESS total score improved significantly in the melatonin arm (mean[SD], total score, Pvalue, 6.854±4.334 ,<0.001). whereas, daytime sleepiness had not significantly changed (mean[SD], total score, *P*value, 13.29±5.11, 0.05).

Certain activities affected by daytime sleepiness, were evaluated by FOSQ-10 and improved in both groups. However, there was a significant difference between arms (-1.630,[CI 95%, -2.401 to -0.835] vs. -0.781[ CI 95%, -1.352 to -0.189] *P*value= 0.006).

### Secondary outcomes

**Fig. 2:**
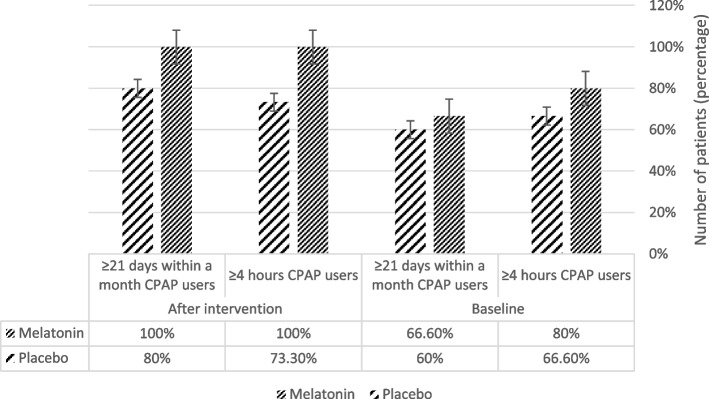
PAP adherence: The data extracted from the PAP devices. Using the device for more than 4 hours during the night and 70% of the duration of the study is considered correct use. The diagram shows the percentage of participants who used PAP appropriately

PAP adherence was measured by the number of nights and hours of PAP usage, extracted from PAP devices. There were 25% and 51% increase in the number of patients using PAP device in the appropriate direction (more than 4 hours and 21 days respectively), in the melatonin arm. Whereas, in the placebo arm this increase in the number of patients were 10% and 33.3%, respectively (Fig. 2). According to the chi-square test, the number of nights using PAP devices was independent of medication use (*P*value=0.068). Whereas, the duration of PAP use during the night, was significantly different between the two groups (*P*-value =0.032).

**Fig. 3:**
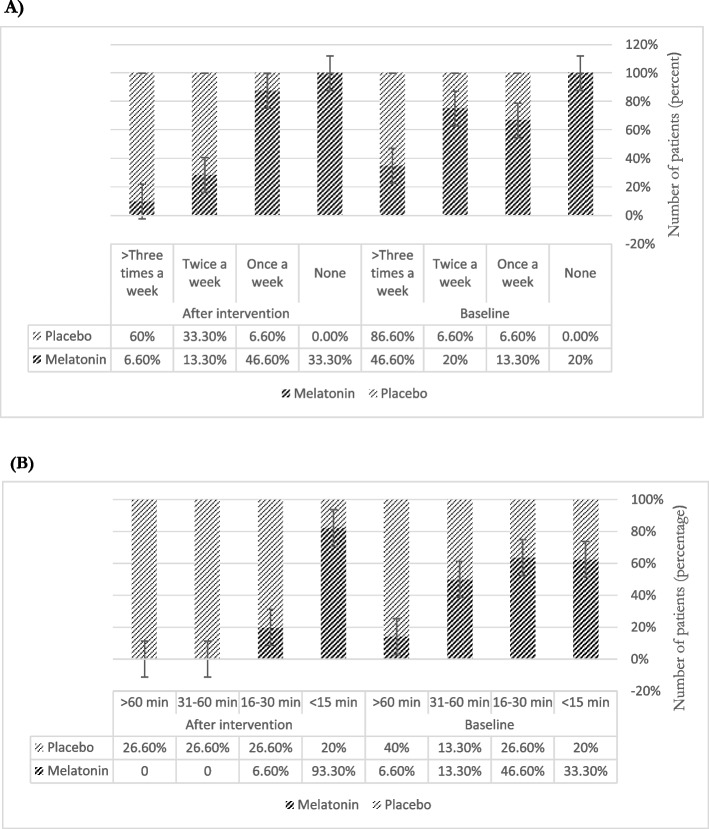
Night-time consequences (PSQI- questionnaire): The data extracted from PSQI- Pittsburgh Sleep Quality Index. The diagrams represent the percentage of patients who had A)Mid-night wake-up B) time to sleep (sleep latency)

Night-time consequences such as sleep latency and midnight wake-up, showed improvements in the melatonin group according to the PSQI questionnaire (Fig. 3). About 33.3% of patients had less than 15 minutes of sleep latency, which improved to 93.3% of patients in the melatonin arm. However, the number of patients with sleep latency of less than 15 minutes, remained unchanged and 66% drop was seen between patients with more than one-hour sleep latency to 31-60 minutes sleep latency. The data was completely dependent on medication use, based on the chi-square test (*P*value<0.001) which represented an improvement in the melatonin arm. Regarding midnight wake-ups, 86.6% of patients woke up more than three nights a week. After the intervention in the placebo arm, this number decreased to 60%. In the melatonin arm, the patients number decreased from 46% to 6.6% (Fig. 3).

Although AHI change was not significantly different between groups. However, AHI in the melatonin arm changed a bit more than placebo group, from the baseline (*P*value = 0.018). There was no significant correlation between intervention and AHI changes (*P*value= 0.683) (Table 3). The total sleep time increased for 8 minutes in the melatonin group. Whereas, this variable increased for about 17 minutes in the placebo group, which was not significantly different (Table 3).

**Table 3:** Secondary outcomes results

	**Arms**						**Mean Difference (SD) (CI 95%)**	***P*value**^**1**^	**Effect size (95% CI)**^**2**^
**Secondary outcomes**	**Melatonin (Mean±SD)**			**Placebo (Mean±SD)**					
	**Before**	**After**	***P* value**^**3**^	**Before**	**After**	***P* value**^**3**^			
**AHI (events/hr.)**	4.91±3.50	3.84±3.54	0.018	5.05±9.25	4.99±9.36	0.937	-1.06(2.66, -6.54 to 4.41)	0.683	1.141 (0.344,1.867)
**Mean total sleep time absolute deviation (hr.)**^**4**^	1.274	1.142	0.434	1.047	1.336	0.166	-	0.769	0.296 (-0.453, 1.037)
**Total sleep time (hr.)**	6.75±1.52	7.64±1.39	-	5.92±1.19	6.46±1.646	**-**	**-**	**-**	0.920(0,130,1.693)
**Sleep efficiency (percent)**^**5**^	79.66± 15.39	91.33± 8.73	0.001	74.33± 18.44	80.40±15.19	0.010	10.93 (4.5, 1.66 to 20.20)	0.011	0.314 (-0.409, 1.039)

^1^ *P* value between groups, Mann–Whitney

^2^ The effect size is based on Cohen's d

^3^ *P*value within group, Wilcoxon test

^4^Mean deviations used to calculate the average deviation from the mean value of total sleep time. It helps to calculate the mean difference between pre-and post-intervention

^5^ Sleep efficiency considered the percentage of time spent asleep while being in bed. It is calculated by the division of the amount of time that patients maintained asleep by the total amount of time stayed in bed. The normal sleep efficiency is more than 85%

Patients who experienced poor sleep quality despite receiving medication during the study were given trazodone tablets at a dose of 25-50 mg, either in the melatonin or placebo group. During the study, six patients received trazodone 25 mg in the placebo group, and five of them received it for more than 12 days. In contrast, only two patients received trazodone in the melatonin group, and for less than five days, particularly in the initial days. According to ANCOVA tests, the usage and medication treatment of Trazodone did not significantly affect total sleep time, sleep quality and daytime sleepiness based on PSQI and ESS (Pvalue>0.001). However, it did have an effect on the FOSQ-10 result in the placebo group. The statistics showed no significant difference in the placebo group from baseline in the absence of Trazodone.

### Safety profile

Regarding adverse effects, follow-up assessments were conducted at 15-day and one-month intervals after the study's completion. If there were any adverse effects, the casualty was assessed by Naranjo score and then documented. During this study, melatonin showed no adverse effects such as drowsiness, headache, fatigue, confusion, or nausea. Daytime sleepiness was not affected by melatonin use.

## Discussion and conclusion

The aim of this study was to evaluate a pharmacologic option at an appropriate dose, 10 mg melatonin, on patients suffering from COMISA who were using PAP devices due to OSA, over a period of one month. Sleep quality, daytime functioning and adherence to PAP therapy were assessed in this randomized double- blind placebo-controlled trial.. According to the results of this clinical trial, a dose of 10 mg of melatonin can improve sleep latency, reduce mid-night wake-ups, and enhance subjective sleep quality, insomnia, and sleep efficiency in COMISA patients, without significantly changing total sleep time compared to the placebo. Moreover, duration of PAP device use was increased based on PAP devices reports which means that adherence to PAP device improved and AHI in melatonin group has significantly changed from the baseline.

Numerous mechanisms can explain the bidirectional relationship between OSA and insomnia. Repetitive respiratory obstructive events and sympathetic activation lead to frequent microarousals, sleep fragmentation and prolonged wakefulness. In addition, nocturia as a consequence of obstructive sleep apnea, results multiple awakenings and difficulty maintaining sleep [34, 35]. On the other hand, insomnia may exacerbate complications of obstructive sleep apnea due to maintaining in bed to prevent refreshment because of awakenings or low arousal threshold. In terms of hormonal release alteration, melatonin nocturnal peak is delayed and shifted to early morning in patients suffering from OSA [36]. In addition, advancing age leads to decrease in melatonin secretion and sleep efficiency. So that, insomnia complications are common in older patients with obstructive sleep apnea [37, 38]. It becomes more complicated if the patients use PAP devices. Positive airway pressure will cause physical discomfort and may be intolerable. As a result, patient compliance with PAP therapy decreases leading to OSA treatment failure, daytime hypersomnolence, poor daytime performance and various comorbidities in long term. Finding an appropriate pharmacological therapy for these patients will be critical improving PAP compliance and sleep quality. According to results of this study, melatonin at the appropriate dose and time, is a safe choice.

It is worthy to compare these results with a parallel group, randomized, double-blind, placebo-controlled pilot effectiveness clinical trial performed by Gooneratne et al. In this study, the efficacy of ramelteon, a melatonin agonist medication, on polysomnographic parameters was considered in elderly patients with COMISA. Twenty-one patients with AHI≥5 received 8 mg ramelteon for four weeks and polysomnography was performed at the initiation and the end of the intervention. This study concluded that ramelteon improve objective sleep quality in patients receiving APAP and suffering from COMISA [39]. Another study about agomelatine, has revealed effectiveness of agomelatine on total sleep time, sleep efficiency and percentage of patients' awakening in the obstructive sleep apnea population [40]. All of these results showed that melatonin and melatonin- based medications may have positive effect on sleep quality with lowest adverse effects in OSA population.

The efficacy of melatonin on comorbid insomnia and other respiratory diseases such as COPD, was assessed in previous studies. Researches showed that 3mg of melatonin could improve subjective sleep quality based on PSQI among these patients. A recent study suggested that melatonin could be an alternative to benzodiazepines for patients suffering from insomnia and COPD [41]. Various clinical trials have investigated the effects of different pharmacotherapy approaches on obstructive sleep apnea. It may be worth considering the short-term use of low-dose doxepin (3-6 mg) and Z-drugs [12]. Sedative-hypnotics, such as eszopiclone, may be used in specific OSA patients with a low to moderate arousal threshold and no significant overnight oxygen desaturation. Furthermore, administering high doses of medications, such as zolpidem, in severe cases of OSA may prolong respiratory events and decrease oxygen levels during the night. However, several studies have shown that sedative-hypnotics do not improve sleep efficiency in patients with OSA [13].

As far as adverse effects are concerned, there was not enough data on melatonin's adverse effects in long term. However, recent studies demonstrate that exogenous melatonin at the dose of 10 mg or more, does not increase serious adverse effects [42–44]. Whereas, other adverse effects such as headaches, especially in the first two days, may occur more [45], so it can be safely administered to the elderly [46]. In the present study, the adverse effects were monitored closely, during and one month after the intervention. The causality was evaluated by the Naranjo score scale. According to the data, no side effects related to melatonin use ,were seen during and after the study.

In conclusion, Melatonin can be a great option in COMISA patients, as it improves sleep quality and increases PAP adherence in this population, without any serious adverse effects. These findings emphasize the importance of considering melatonin as part of a comprehensive treatment plan for patients with COMISA. Further research is necessary to evaluate long-term effects or basic studies should be conducted to find out the mechanism of melatonin in such population. Nonetheless, melatonin represents a valuable tool in improving the sleep quality and overall well-being of individuals with COMISA.

### Limitations

- As the overall prevalence of COMISA is low and the price of PAP devices increased throughout the study, the population was restricted. Despite not reaching the total sample size calculated at the initiation of the study due to some limitations, the sample power was more than 90% for primary outcomes
- PAP device reports demonstrate mean nighttime PAP use by hours and number of nights, during a month. So, the exact time of sleep and wake-ups were not measured. In order to find out the duration of sleep, polysomnography could be applied before and after the intervention.
- Even though some patients needed to take other hypnotics during the study, the results showed that using hypnotics does not affect the total sleep time and sleep quality, in both groups.

## Data Availability

The datasets used and/or analyzed during the current study are available from the corresponding author upon reasonable request.

## Abbreviations

AHI: Apnea- Hypopnea Index
ANCOVA: Analysis of Covariance
BMI: Body Mass Index
COMISA: Comorbid Insomnia and Obstructive Sleep Apnea
COPD: Chronic Obstructive Pulmonary Disease
ESS: Epworth sleepiness scale
FOSQ-10: Functional Outcomes of Sleep Questionnaire (FOSQ-10)
GOLD: Global Initiative for Chronic Obstructive Lung Disease
ICSD-3: The International Classification of Sleep Disorders – Third Edition
ILD: Interstitial lung diseases
IRCT: Iranian Registry of Clinical Trials
ISI: Insomnia Severity Index
NYHA: The New York Heart Association
OSA: Obstructive Sleep Apnea
PAP: Positive Airway Pressure
PMH: Past Medical History
PSQI: The Pittsburgh Sleep Quality Index
